# *Cryptosporidium mortiferum* n. sp. (Apicomplexa: Cryptosporidiidae), the species causing lethal cryptosporidiosis in Eurasian red squirrels (*Sciurus vulgaris*)

**DOI:** 10.1186/s13071-023-05844-8

**Published:** 2023-07-15

**Authors:** Lenka Tůmová, Jana Ježková, Jitka Prediger, Nikola Holubová, Bohumil Sak, Roman Konečný, Dana Květoňová, Lenka Hlásková, Michael Rost, John McEvoy, Lihua Xiao, Monica Santín, Martin Kváč

**Affiliations:** 1 Faculty of Agriculture and Technology, University of South Bohemia in České Budějovice, Studentská 1668, 37005 České Budějovice, Czech Republic; 2 Institute of Parasitology, Biology Centre of the Czech Academy of Sciences, Branišovská 31, 370 05 České Budějovice, Czech Republic; 3 Microbiological Sciences Department, North Dakota State University, 1523 Centennial Blvd, Van Es Hall, Fargo, ND 58102 USA; 4 Center for Emerging and Zoonotic Diseases, College of Veterinary Medicine, South China Agricultural University, Guangzhou, 510642 Guangdong China; 5 Guangdong Laboratory for Lingnan Modern Agriculture, Guangzhou, 510642 Guangdong China; 6 Environmental Microbial and Food Safety Laboratory, Beltsville Agricultural Research Center, Agricultural Research Service, US Department of Agriculture, Beltsville, MD USA

**Keywords:** Mortality, Biology, Course of infection, Cryptosporidiosis, Oocyst size, Phylogeny, Genetic diversity

## Abstract

**Background:**

*Cryptosporidium* spp. are globally distributed parasites that infect epithelial cells in the microvillus border of the gastrointestinal tract of all classes of vertebrates. *Cryptosporidium* chipmunk genotype I is a common parasite in North American tree squirrels. It was introduced into Europe with eastern gray squirrels and poses an infection risk to native European squirrel species, for which infection is fatal. In this study, the biology and genetic variability of different isolates of chipmunk genotype I were investigated.

**Methods:**

The genetic diversity of *Cryptosporidium* chipmunk genotype I was analyzed by PCR/sequencing of the *SSU rRNA*, *actin*, *HSP70*, *COWP*, *TRAP-C1* and *gp60* genes. The biology of chipmunk genotype I, including oocyst size, localization of the life cycle stages and pathology, was examined by light and electron microscopy and histology. Infectivity to Eurasian red squirrels and eastern gray squirrels was verified experimentally.

**Results:**

Phylogenic analyses at studied genes revealed that chipmunk genotype I is genetically distinct from other *Cryptosporidium* spp. No detectable infection occurred in chickens and guinea pigs experimentally inoculated with chipmunk genotype I, while in laboratory mice, ferrets, gerbils, Eurasian red squirrels and eastern gray squirrels, oocyst shedding began between 4 and 11 days post infection. While infection in mice, gerbils, ferrets and eastern gray squirrels was asymptomatic or had mild clinical signs, Eurasian red squirrels developed severe cryptosporidiosis that resulted in host death. The rapid onset of clinical signs characterized by severe diarrhea, apathy, loss of appetite and subsequent death of the individual may explain the sporadic occurrence of this *Cryptosporidium* in field studies and its concurrent spread in the population of native European squirrels. Oocysts obtained from a naturally infected human, the original inoculum, were 5.64 × 5.37 μm and did not differ in size from oocysts obtained from experimentally infected hosts. *Cryptosporidium* chipmunk genotype I infection was localized exclusively in the cecum and anterior part of the colon.

**Conclusions:**

Based on these differences in genetics, host specificity and pathogenicity, we propose the name *Cryptosporidium mortiferum* n. sp. for this parasite previously known as *Cryptosporidium* chipmunk genotype I.

**Graphical Abstract:**

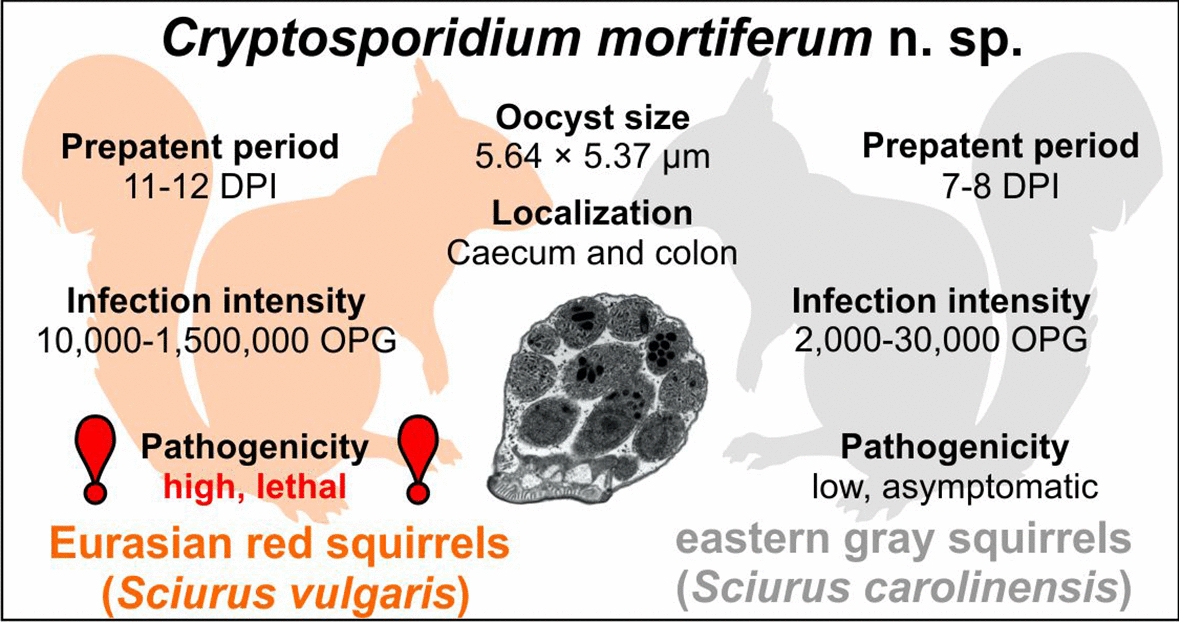

## Background

*Cryptosporidium* spp. are important causative agents of mild to severe diarrheal diseases in humans and various animals, with worldwide distribution [1]. The first descriptions of *Cryptosporidium* species were based on descriptions of oocyst morphology, localization of infection in the host or the occurrence in hosts [2–4]. For this reason, few species had been identified within the genus *Cryptosporidium* by the end of the twentieth century, with most species belonging to the *Cryptosporidium parvum*- and *C. muris*-like complexes [5, 6]. Developments in molecular biology and routine use of molecular techniques in research have contributed significantly to the discovery of great genetic diversity within the genus *Cryptosporidium* and supported the description of many new species and genotypes [7–10]. To date, there are 49 established species and dozens of genotypes that have been described based on molecular differences from valid species [7, 11]. Some species and genotypes, such as *C. parvum*, *C. hominis*, *C. meleagridis*, *C. felis* and *C. viatorum*, frequently cause diarrheal disease in humans, livestock and wildlife [3, 12–14], while others are not associated with clinical disease [15–17]. We have little knowledge about the biology and course of infection of most *Cryptosporidium* genotypes, but given their genetic divergence from named species, they likely represent distinct species.

*Cryptosporidium* chipmunk genotype I was first described in 2005 as *Cryptosporidium* isolate W17 in surface waters in the USA [18]. In 2007, the same genotype was identified in rodent fecal samples from the watershed of the New York City water supply, and it was renamed chipmunk genotype I [19]. Molecular epidemiological studies have shown that the natural hosts of chipmuk genotype I in North America are mainly eastern chipmunks (*Tamias striatus*) and eastern gray squirrels (*Sciurus carolinensis*) and occasionally rodents of the genus *Peromyscus* [19, 20]. In Europe, chipmunk genotype I was first reported in two Eurasian red squirrels (*Sciurius vulgaris*) in Italy [21]. Eastern gray squirrels and native Eurasian red squirrels are sympatric species in the UK and Italy [22, 23], most likely due to the introduction of the eastern gray squirrel into Europe during the last century. Prediger et al. [24] demonstrated that eastern gray squirrels introduced in northern Italy are parasitized with chipmunk genotype I. However, chipmunk genotype I was not detected in the Eurasian red squirrels in the same study, and therefore it was concluded that this genotype rarely infects them. In 2021, natural infections with chipmunk genotype I were reported in sick Eurasian red squirrels housed at a rehabilitation center in Sweden, one of which subsequently died of intestinal disease [25]. Based on these data, we hypothesized that chipmunk genotype I is highly pathogenic to Eurasian red squirrels, causing frequent mortality, which could explain why it was not detected in surveys of wild Eurasian red squirrels. The aim of this study was to describe the infectivity and pathogenicity of chipmunk genotype I in Eurasian red squirrels and eastern gray squirrels. Morphological and genetic characteristics of this *Cryptosporidium* genotype were studied. Findings demonstrated that chipmunk genotype I is highly pathogenic to the Eurasian red squirrel. Moreover, the data obtained confirmed that chipmunk genotype I is genetically and biologically distinct from valid *Cryptosporidium* species, and therefore we propose to name it *Cryptosporidium mortiferum* n. sp.

## Methods

### Source of chipmunk genotype I oocysts

The isolate (Chip_I) of chipmunk genotype I was obtained from a naturally infected, immunocompetent adult living in the USA. However, the patient’s residency information is not available. The presence of oocysts in the diarrheal stool specimen was detected by direct immunofluorescence assay (Merifluor; Meridian Biosciences, Cincinnati, Ohio, USA), and the genotype was determined by PCR/sequencing. Oocysts from isolate Chip_I, purified by cesium chloride gradient centrifugation [26], were used for morphometric, molecular characterization and cross-transmission studies. In addition to isolate Chip_I, DNA from an additional five isolates (14762, 17064, 15003, SV33 and SV59), obtained previously from naturally infected eastern gray squirrels, Pallas squirrels (*Callosciurus erythraeus*) and Eurasian red squirrels in Italy, were used for molecular characterization [21, 24].

### Oocyst morphometry

Oocysts of chipmunk genotype I (isolate Chip_I), from a naturally infected human and from experimentally infected hosts in the present study, were purified by cesium chloride [26] and used for morphological analyses. The length and width of 100 oocysts of each isolate were measured, and a shape index was calculated. Pure oocysts from the *C. parvum* isolate HA (*n* = 100), originally obtained from a naturally infected calf and maintained in SCID mice in the laboratories of the Biology Center of the Czech Academy of Science, Czech Republic (BC CAS), were used as controls. Oocysts were measured by the same worker using differential interference contrast (DIC) microscopy at 1000× magnification. Each slide was analyzed in a meandering pattern to avoid repeated measurement of an oocyst. Photographs of oocysts for morphometric analysis were analyzed using digital image analysis (Olympus CellSens Entry 2.1 software and Olympus Digital DP73 color camera, Olympus Corporation, Shinjuku, Tokyo, Japan). Various staining and labeling methods were also used to visualize the oocysts. Fecal samples containing oocysts of chipmunk genotype I were stained with aniline-carbol-methyl violet (ACMV) [27], modified Ziehl-Neelsen (ZN) [28] and phenol stain (AP) [29] and labeled with genus-specific FITC-conjugated antibodies (IFA; *Cryptosporidium* IF test, Cryptocell, Cellabs Pty Ltd., Brookvale, Australia) and with a Cy3-labeled mouse monoclonal antibody targeting the antigenic sites of the *Cryptosporidium* oocyst outer wall (A400Cy2R-20X, Crypt-a-Glo, Waterborne, Inc., New Orleans, LA, USA).

### Molecular characterization and sequencing

Total genomic DNA (gDNA) was extracted from purified oocysts (100,000), feces (200 mg) or tissue samples (200 mg) using a GeneAll^®^ Exgene™ Stool DNA Mini Kit (GeneAll Biotechnology Co., Ltd.; Seoul, South Korea) or a DNeasy Blood & Tissue Kit (QIAGEN, Hilden, Germany) according to the manufacturer’s instructions, followed by bead disruption of the oocysts for 60 s at 5.5 m/s with 0.5-mm glass beads in a FastPrep^®^-24 Instrument (MP Biomedicals, Santa Ana, CA, USA). The acquired gDNA was stored at − 80 °C. The partial sequences of the genes for the small subunit of rRNA (*SSU*), actin, *Cryptosporidium* oocyst wall protein (*COWP*), 70-kDa heat shock protein (*HSP70*), thrombospondin-related adhesive protein of *Cryptosporidium*-1 (*TRAP-C1*) and 60-kDa glycoprotein (*gp60*) were amplified according to published nested PCR protocols and PCR primers [18, 30–34]. Primary and secondary reactions were performed in a 50 µl volume. The primary mixture consisted of 2 μl gDNA, 2.5 U Taq DNA polymerase (Dream Taq Green DNA Polymerase, Thermofisher Scientific, Waltham, MA, USA), 1× PCR buffer (Thermofisher Scientific), 200 nM of each primer, 6 mM MgCl_2_ (*SSU*) or 3 mM MgCl_*2*_ (actin, *COWP*, *HSP70*, *TRAP-C1* and *gp60*), 200 uM of each deoxynucleoside triphosphate and 2 ul of nonacetylated bovine serum albumin (BSA; 10 mg/ml; New England Biolabs, Beverly, MA, USA) and molecular grade water. The mixtures for secondary PCR were similar to those described above for primary PCR, except that 2 μl of the primary PCR product was used as the template, and the concentration of MgCl_2_ for all amplified genes was 3 mM. Molecular water was used as a negative control, and DNA from *Cryptosporidium tyzzeri* (for *SSU*, actin, *COWP*, *HSP70* and *TRAP-C1*) and chipmunk genotype I (for *gp60*) were used as positive controls. Secondary PCR products were separated by electrophoresis in a 2% agarose gel and stained with ethidium bromide. The amplicons were purified using the GenElute™ Gel Extraction Kit (Sigma, St. Louis, MO, USA) and sequenced using the secondary PCR primers by the commercial company SeqMe, s.r.o. (Dobříš, Czech Republic).

### Phylogenetic analysis

The nucleotide sequences obtained were edited using Chromas Pro 2.4.1 software (Technelysium, Pty, Ltd., South Brisbane, Australia), verified by BLAST analysis (https://blast.ncbi.nlm.nih.gov/Blast.cgi), edited and aligned using BioEdit v.7.0.5 (Hall 1999). Final alignment of the obtained sequences with the reference sequences from GenBank was performed using the online server MAFFT version 7 (http://mafft.cbrc.jp/alignment/software/). The best model for the DNA/protein phylogeny was selected for each alignment using the Bayesian information criterion in MEGA 7. Neighbor-joining (NJ) and maximum likelihood (ML) approaches were computed in MEGA7 software [35, 36], using Tamura’s three-parameter model + G + I [37] for the *SSU*, actin and *COWP* alignment and the general time-reversible model + G + I [38] for the *gp60*, *HSP70* and *TRAP-C1* alignment. Bootstrap support for branching was based on 1000 replications. Final trees were visualized using Corel Draw X7 software (https://www.corel.draw.com). The sequences obtained in this study were deposited in GenBank under the following accession numbers: OQ627025 to OQ627029 (*SSU*), OQ632461 to OQ632466 (actin), OQ632467 to OQ632474 (*COWP*), OQ632480 to OQ632487 (*HSP70*), OQ632488 to OQ632495 (*TRAP-C1*) and OQ632475 to OQ632479 (*gp60*).

### Transmission studies

Fecal samples from all animals used in the transmission studies were examined daily for the presence of *Cryptosporidium* spp. oocysts and specific DNA (*SSU*) for 1 week prior to the transmission studies. A single 8-week-old SCID mouse (SCID 0) was infected with a dose of 100,000 oocysts of chipmunk genotype I isolate Chip_I. The oocysts of chipmunk genotype I obtained from SCID 0 were compared morphologically and molecularly with the original isolate Chip_I and used to infect other animals (see below). Three 8-week-old mice (*Mus musculus*) of each strain (BALB/c, SCID, C57Bl6, CD4^−/−^ and CD8^−/−^), three 8-week-old gerbils (*Meriones unquiculatus*), three adult Guinea pigs (*Cavia aperea*), three adult ferrets (*Mustela putorius furo*), three adult Eurasian red squirrels, three adult eastern gray squirrels and three 7-day-old chickens (*Gallus gallus* f. *domestica*) were used for experimental transmission studies. Three animals from each group served as negative controls. Each experimental animal was orally administered a dose of 100,000 oocysts of chipmunk genotype I in a 200 μl volume. Control groups were orally inoculated with 200 μl sterile water. Beginning on the second day after inoculation, feces from all animals were collected individually and examined for the presence of oocysts (ACMV stain) and specific *Cryptosporidium* DNA (*SSU*). All animals were monitored for 30 days post-infection (DPI) or less if their health deteriorated because of infection and were therefore humanely killed. Oocysts were quantified, and the intensity of infection was estimated using the method described by Kváč et al. [20]. Briefly, the slide was weighed to the nearest 0.001 g before and after preparation of the smear to determine the mass of fecal material added to the slide. After ACMV staining, all oocysts on the slide were counted, and the number of oocysts per gram of fecal material was calculated. Oocysts were counted from triplicate smears of each sample. All experimental procedures complied with the laws of the Czech Republic (Act No. 246/1992 Coll., on the Protection of Animals against Cruelty, under protocols nos. MZP/2019/630/1411 and 35/2018). Rodents were housed individually in ventilated cages (Tecniplast, Buguggiate, Italy). Chickens were housed in boxes. Squirrels and ferrets were housed in separate cages. An external heat source was used for young birds during the first 5 days of life. Sterilized food and water were available ad libitum for all animals. Keepers wore sterile shoe covers, disposable coveralls and disposable gloves when entering the experimental room. Wood chip bedding and disposable protective clothing were removed from the experimental room and incinerated.

### Clinical and pathomorphological examinations

All animals that shed oocysts or specific DNA after 30 DPI or that were killed early were necropsied. Tissue samples from the esophagus, stomach (mammalian only), proventriculus and ventriculus (chicken only), duodenum, proximal, central and distal jejunum; ileum, cecum, colon, cloaca and bursa of Fabricius (chicken only), liver, spleen, kidney, urinary bladder, trachea, lung, heart, eye and brain were collected using different sterile dissection tools for each site and processed for histology, scanning and transmission electron microscopy (SEM and TEM) and PCR genotyping (see above). Histological sections (5 μm) were stained with hematoxylin and eosin (H&E) and periodic acid-Schiff (PAS) and examined at 100–400× magnification (Olympus IX70) [39]. Samples for SEM were processed according to the protocol in Kváč et al. [15] and viewed using a JEOL JSM-7401F-FE SEM. Samples for TEM were processed according to the protocol in Valigurová et al. [40] and viewed using a JEOL JEM-2100F.

### Staining of mucosal smears

Identification of developmental stages in the gastrointestinal tract of SCID mice was performed using Wright stained smears, which allow visualization of characteristic morphological structures [2]. Tissue samples from the cecum (selected based on the results of PCR, histology, SEM and TEM) were carefully washed with cold sterile PBS. The washed tissue was exposed to serum from *Cryptosporidium*-negative mice for 5 min to release developmental stages. The mucosa was carefully scraped with a scalpel and smeared on a glass. The wet smears were fixed in the vapor of 2% osmium tetroxide for 30 min and washed with methanol. The fixed smears were stained for 9 min with Wright diluted 1:1 with distilled water. Sporozoites released from excysted oocysts were stained with carbol-fuchsin [27]. Slides were viewed at 1000× magnification and documented using Olympus cell Sens Entry 2.1 (Olympus Corporation, Shinjuku, Tokyo, Japan) and a digital camera (Olympus DP73).

### Statistical analysis

Differences in *Cryptosporidium* spp. oocyst size were tested using Hotelling’s multivariate version of the two sample *t*-test, package ICSNP: Tools for Multivariate Nonparametrics [41] in R 4.2.2. [42]. The hypothesis tested was that the two-dimensional mean vectors of measurement are the same in the two populations being compared.

## Results

### Sequence and phylogenetic analysis

**Fig. 1:**
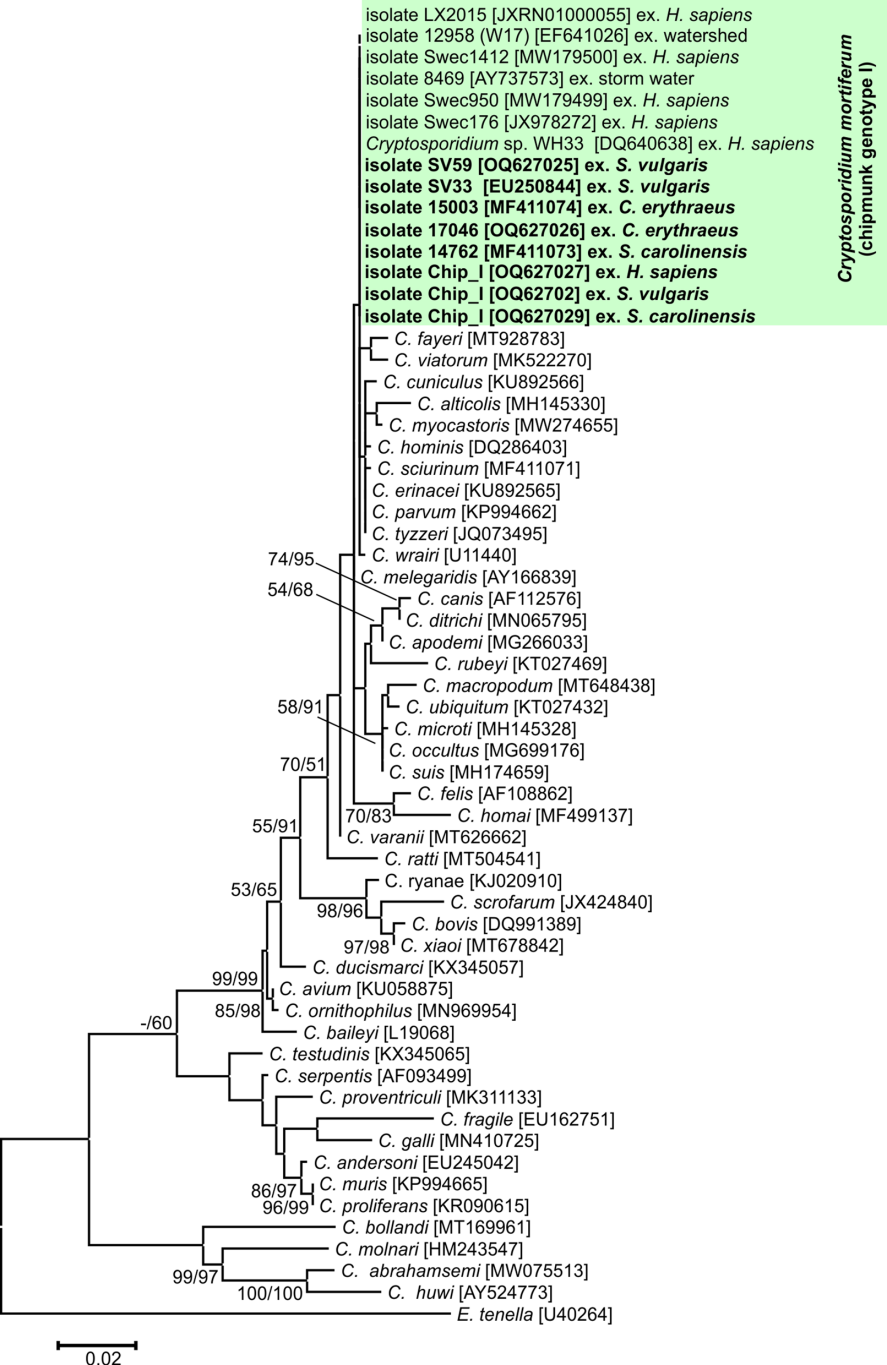
Evolutionary relationships of *Cryptosporidium* spp. at the small subunit rRNA locus (*SSU*) inferred using the maximum likelihood (ML)/neighbor-joining (NJ) method. Percentage supports (> 50%) from 1000 pseudoreplicates from ML and NJ analysis, respectively, are indicated next to supported node. The GenBank accession number is in parentheses. Sequences obtained in this study are identified by isolate number (e.g. 14762) and highlighted

**Fig. 2:**
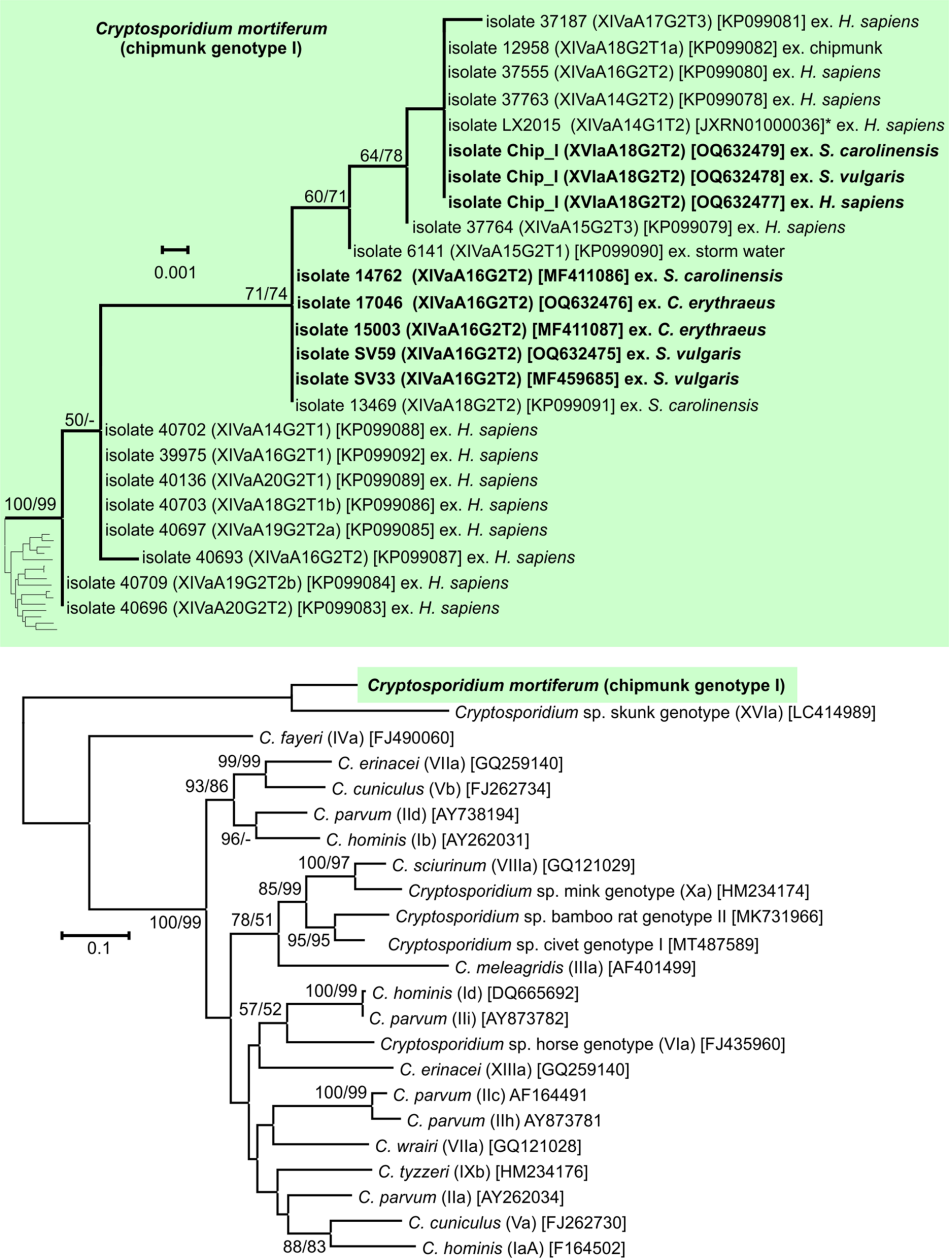
Evolutionary relationships of *Cryptosporidium* spp. at the 60 kDa glycoprotein locus (gp60) inferred using the maximum likelihood (ML)/neighbor-joining (NJ) method. Percentage supports (> 50%) from 1000 pseudoreplicates from ML and NJ analysis, respectively, are indicated next to the supported node. The GenBank accession number is in parentheses. Sequences obtained in this study are identified by isolate number (e.g. 14762) and highlighted

**Fig. 3:**
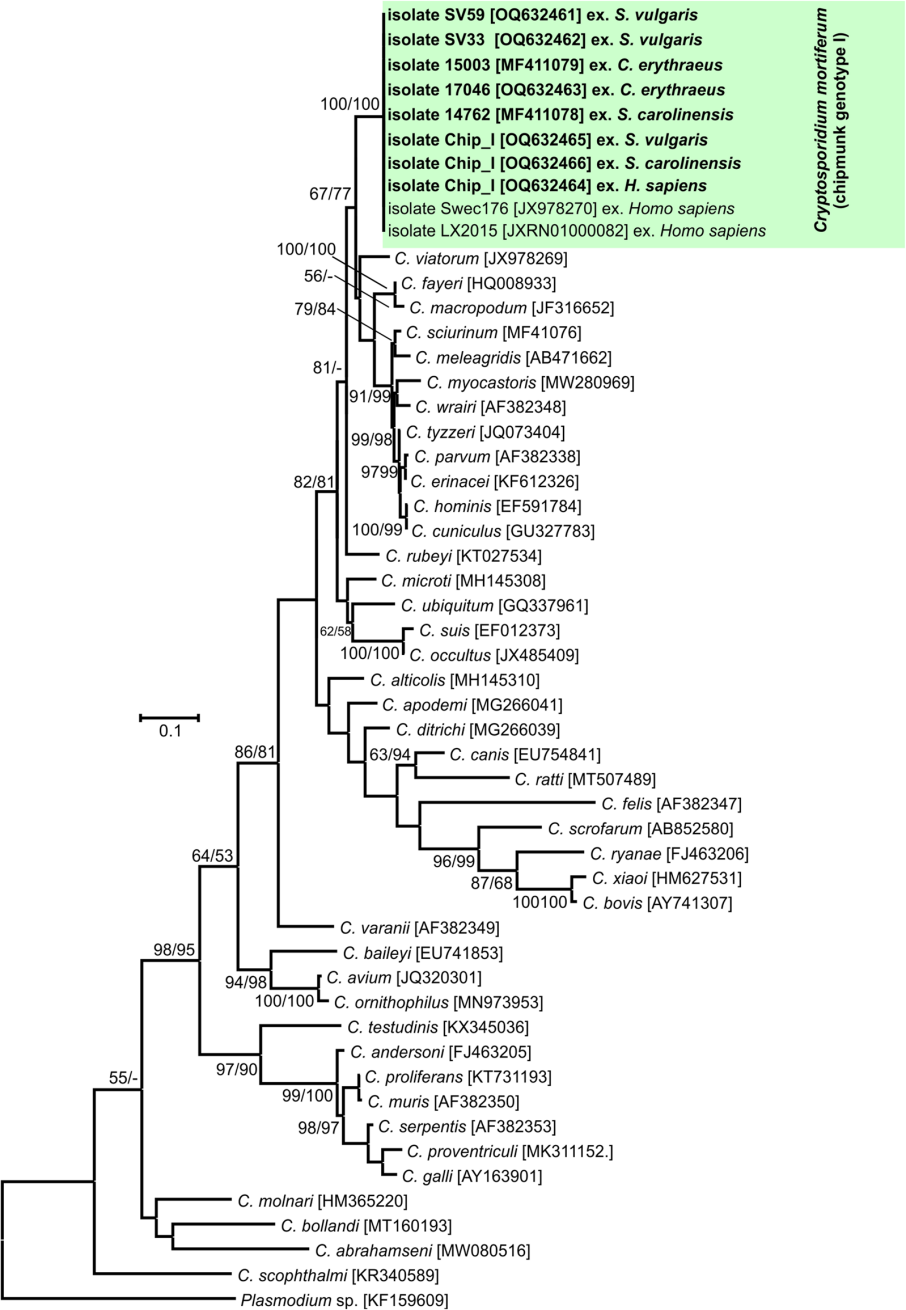
Evolutionary relationships of *Cryptosporidium* spp. at the actin locus inferred using the maximum likelihood (ML)/neighbor-joining (NJ) method. Percentage supports (> 50%) from 1000 pseudoreplicates from ML and NJ analysis, respectively, are indicated next to supported node. The GenBank accession number is in parentheses. Sequences obtained in this study are identified by isolate number (e.g. 14762) and highlighted

**Fig. 4:**
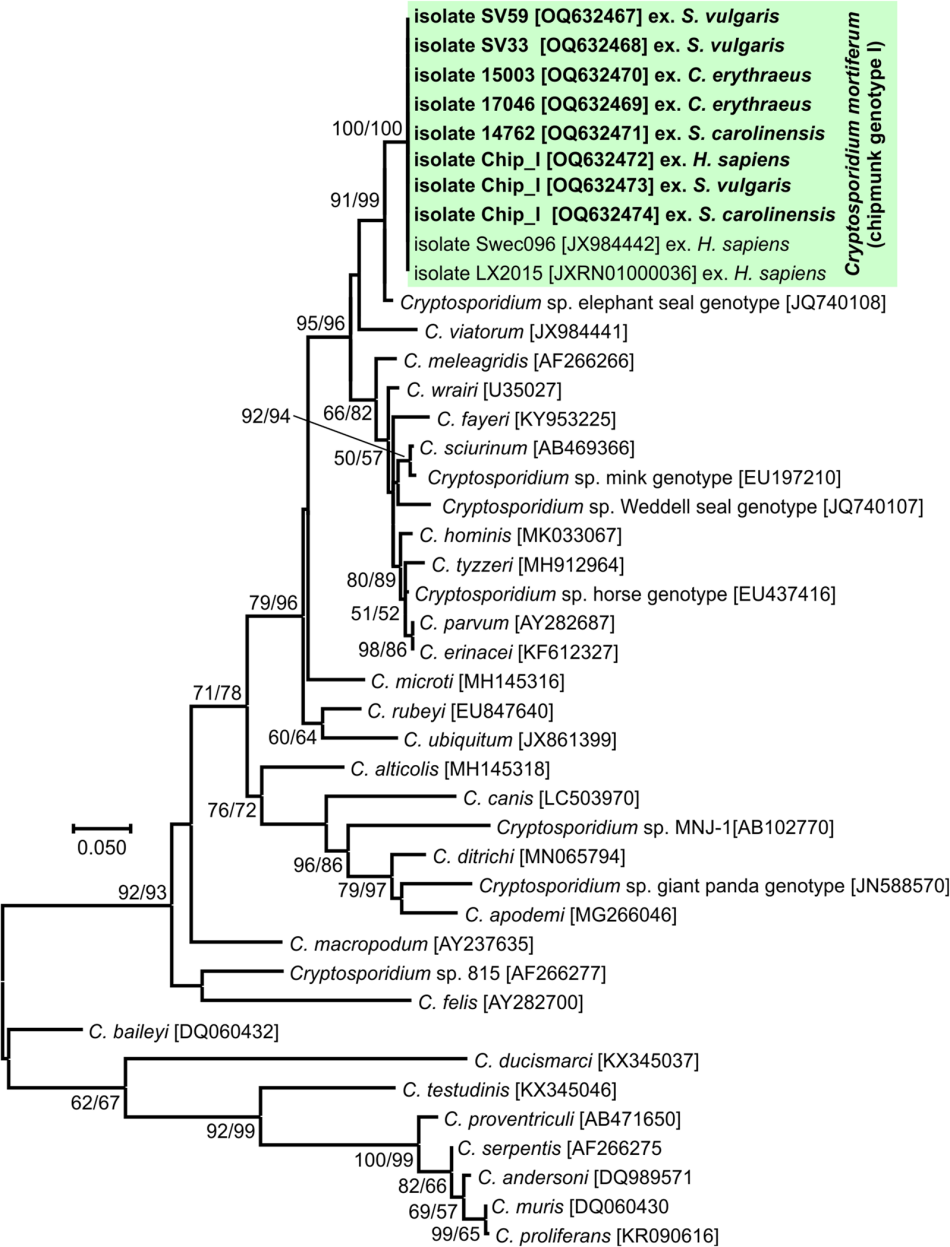
Evolutionary relationships of *Cryptosporidium* spp. at the *Cryptosporidium* oocyst wall protein (*COWP*) locus inferred using the maximum likelihood (ML)/neighbor-joining (NJ) method. The evolutionary distances were computed using the General Time Reversible model with a gamma distribution. Percentage supports (> 50%) from 1000 pseudoreplicates from ML and NJ analysis, respectively, are indicated next to supported node. The GenBank Accession number is in parentheses. Sequences obtained in this study are identified by isolate number (e.g. 14762) and highlighted

**Fig. 5:**
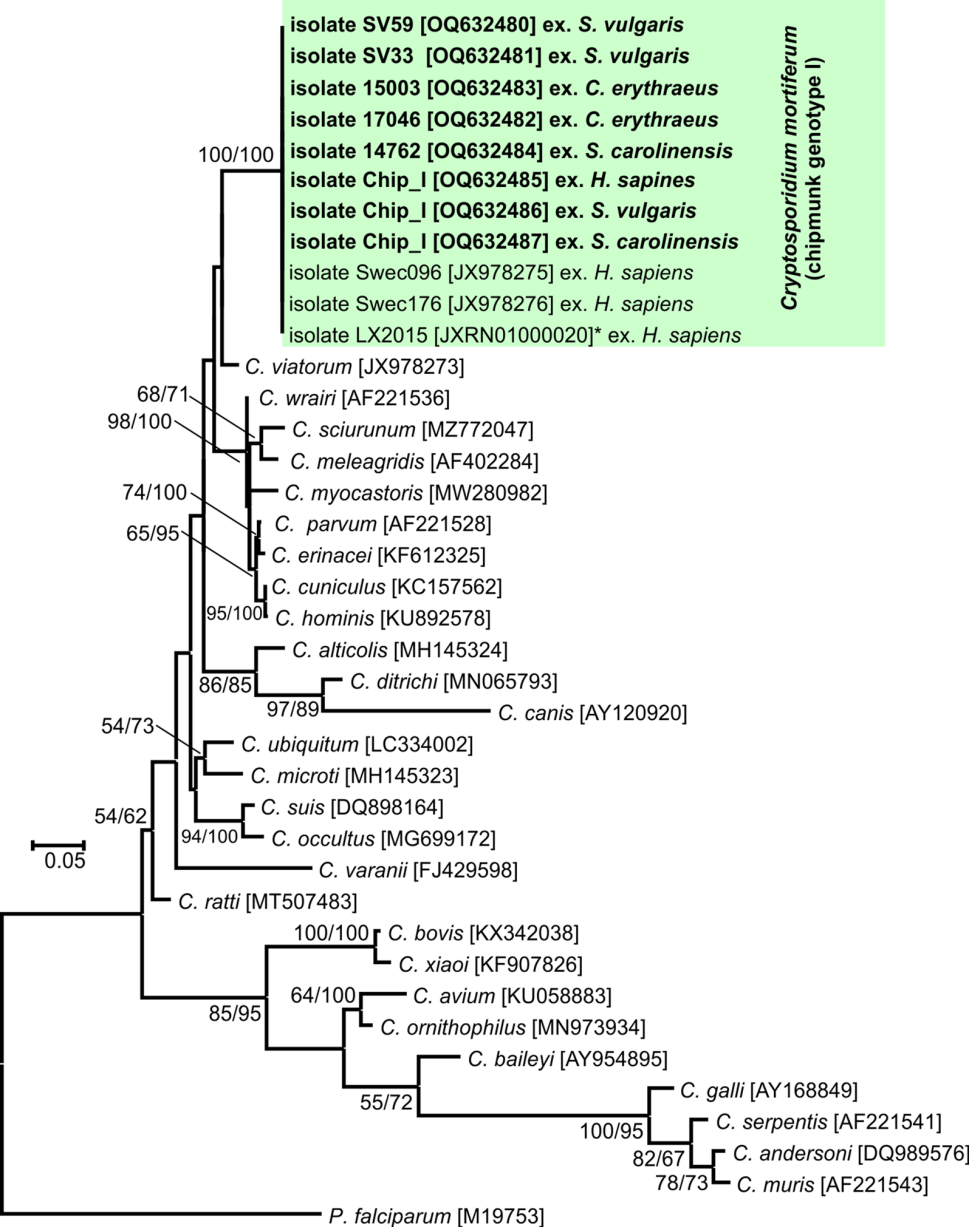
Evolutionary relationships of *Cryptosporidium* spp. at the 70-kDa heat shock protein (*HSP70*) inferred using the maximum likelihood (ML)/neighbor-joining (NJ) method. Percentage supports (> 50%) from 1000 pseudoreplicates from ML and NJ analysis, respectively, are indicated next to supported node. The GenBank Accession number is in parentheses. Sequences obtained in this study are identified by isolate number (e.g. 14762) and highlighted

**Fig. 6:**
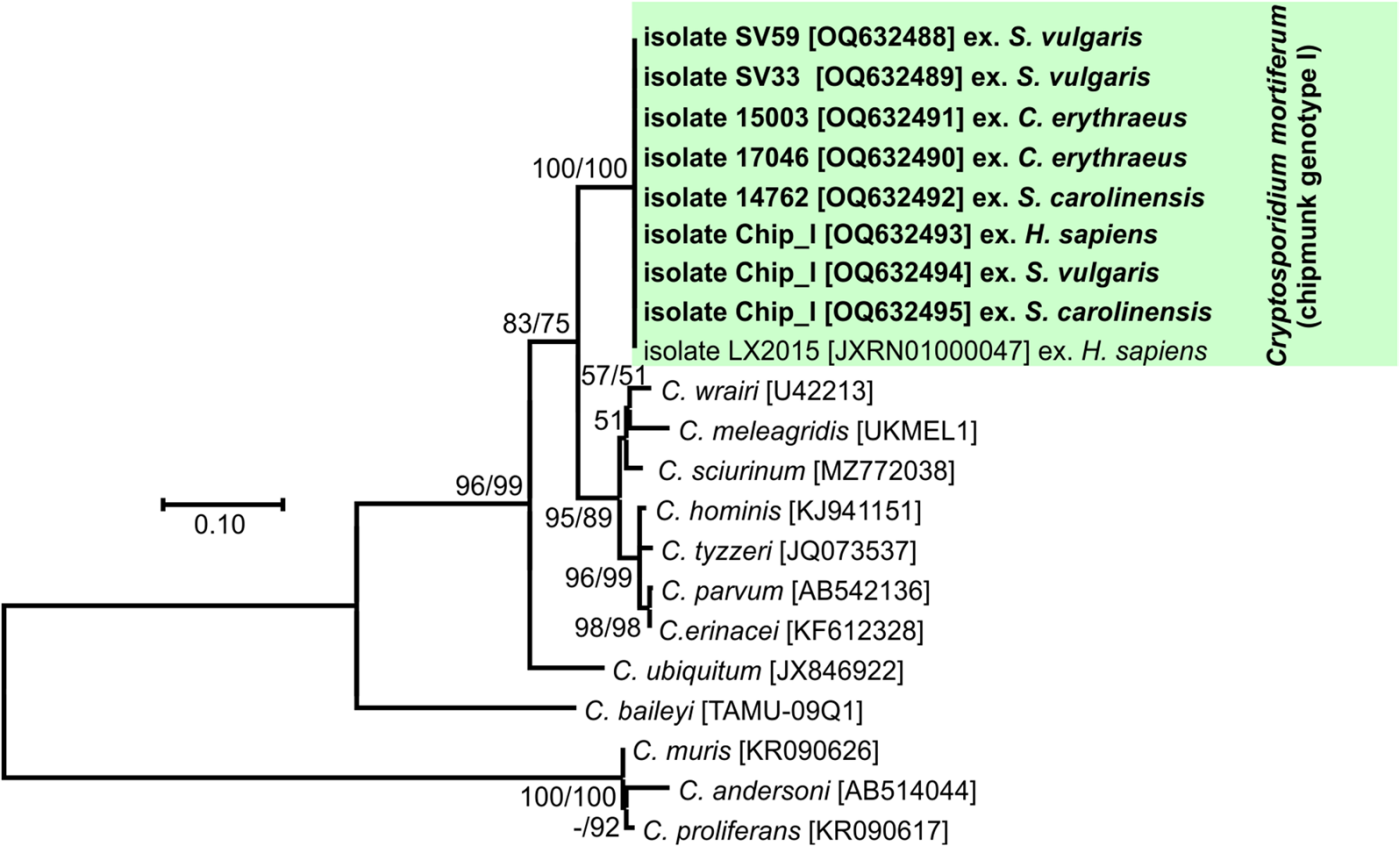
Evolutionary relationships of *Cryptosporidium* spp. at the thrombospondin-related adhesive protein of *Cryptosporidium*-1 (*TRAP-C1*) locus inferred using the maximum likelihood (ML)/neighbor-joining (NJ) method. Percentage supports (> 50%) from 1000 pseudoreplicates from ML and NJ analysis, respectively, are indicated next to supported node. The GenBank accession number is in parentheses. Sequences obtained in this study are identified by isolate number (e.g. 14762) and highlighted

Partial amplicons of the genes encoding *SSU*, actin, *COWP*, *HSP70*, *TRAP-C1* and *gp60* were obtained from all chipmunk genotype I isolates included in this study from naturally infected Eurasian red squirrels (SV33 and SV59), eastern gray squirrels (14762), Pallas squirrels (15003 and 17064), a human (isolate CHIP_I) and experimentally infected laboratory mice, gerbils, ferrets, Eurasian red squirrels and eastern gray squirrels (isolate CHIP_I) (Figs. 1, 2, 3, 4, 5 and 6). The sequences of individual genes obtained from experimentally infected animals were not different from each other and were also identical to the original isolate CHIP_I. Phylogenetic relationships among chipmunk genotype I isolates and other *Cryptosporidium* spp. were inferred by ML and NJ analyses at all six loci and revealed similar tree topologies. All isolates of chipmunk genotype I used in this study were identical to each other and clustered with other previously reported chipmunk genotype I isolates (Figs. 1, 2, 3, 4, 5 and 6).

### Host specificity and course of infection

**Fig. 7:**
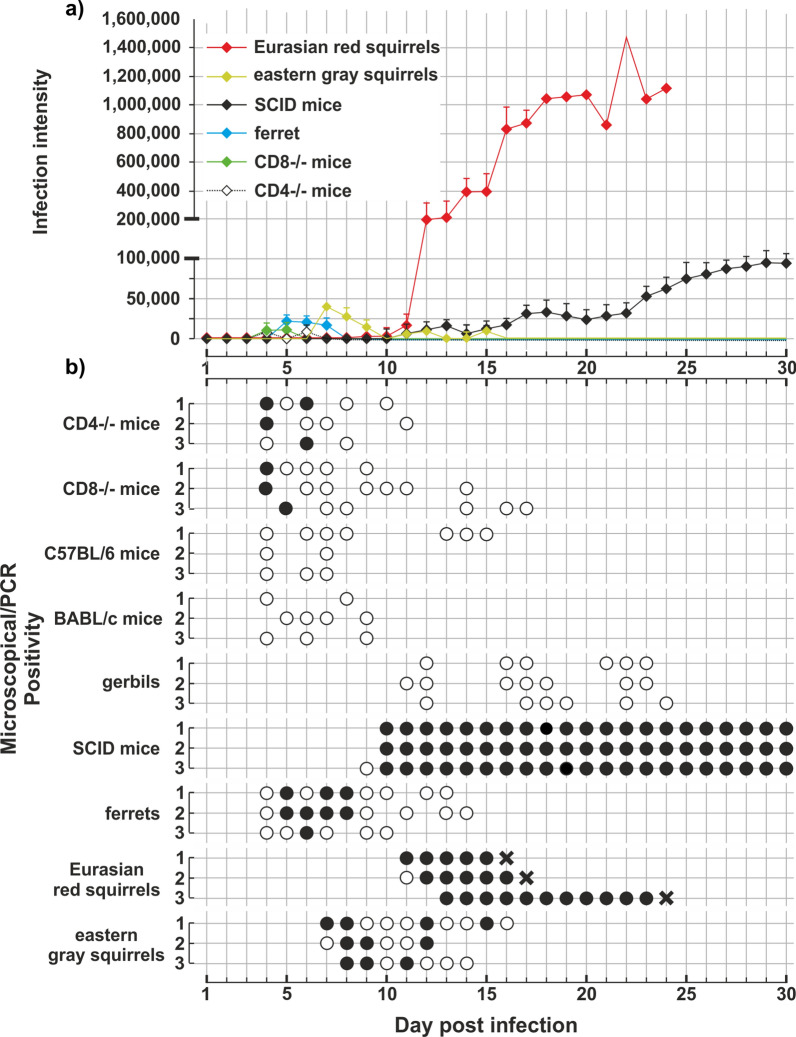
Course of infection of *Cryptosporidium* chipmunk genotype I. in different strains of experimentally inoculated laboratory mice (*Mus musculus*), gerbils (*Meriones unquiculatus*), ferrets (*Mustela putorius furo*), Eurasian red squirrels (*Sciurus vulgaris*) and eastern gray squirrels (*Sciurus carolinensis*). **a** Infection intensity expressed as number of oocysts per gram of feces (OPG) and **b** detection of oocysts based on molecular and microscopic examination of fecal samples. Black circles indicate the presence of oocysts and specific DNA of *Cryptosporidium* chipmunk genotype I; white circles indicate detection of specific-DNA only without oocyst detection. Crosses indicate that an animal was killed because of poor health

No infection was detectable in chickens and Guinea pigs inoculated with 100,000 chipmunk genotype I oocysts (data not shown). Eurasian red squirrels, eastern gray squirrels, gerbils, ferrets and all mouse strains were susceptible to infection with chipmunk genotype I. In adult BALB/c and C57Bl6 mice, infection was not detectable microscopically, but molecular analyses revealed repeated presence of chipmunk genotype I DNA in fecal samples starting at 4–5 DPI (Fig. 7). CD4^−/−^ and CD8^−/−^ mice shed microscopically detectable oocysts with an intensity of 2000–6000 OPG starting at 4–5 DPI. Infection in gerbils was detected by PCR only with the prepatent period was determined to be 11–12 DPI, and subsequently chipmunk genotype I DNA was detected intermittently until day 24 (Fig. 7b). Ferrets began shedding oocysts at 4 DPI. The intensity of infection ranged from 2000 to 20,000 OPG (Fig. 7a), and ferrets recovered spontaneously at 10–14 DPI. In SCID mice, the prepatent period was 9–10 DPI, and the animals shed many oocysts daily (10,000–150,000 OPG), without spontaneous recovery during the experiment (30 DPI). The prepatent period in eastern gray squirrels (7–8 DPI) was almost half that in Eurasian red squirrels (11–12 DPI). While eastern gray squirrels shed oocysts intermittently, with infection intensity ranging from 2000 to 30,000 OPG, infection in Eurasian red squirrels ranged from 10,000 to 1,500,000 OPG. In one of the eastern gray squirrels, mild apathy was observed at 7–8 DPI, manifested by decreased interest in the environment and food. The feces of this individual had a pasty consistency during this time. All eastern gray squirrels lost the infection within 9 days of the onset of shedding. Eurasian red squirrels rapidly lost their condition and appetite for food beginning on day 10 after infection. They spent most of the day in their shelter, were lethargic and did not respond to external stimuli such as feeding, watering, handling or cage cleaning. All Eurasian red squirrels with severe clinical signs were humanely killed (Fig. 7b).

### Oocyst morphology

**Fig. 8:**
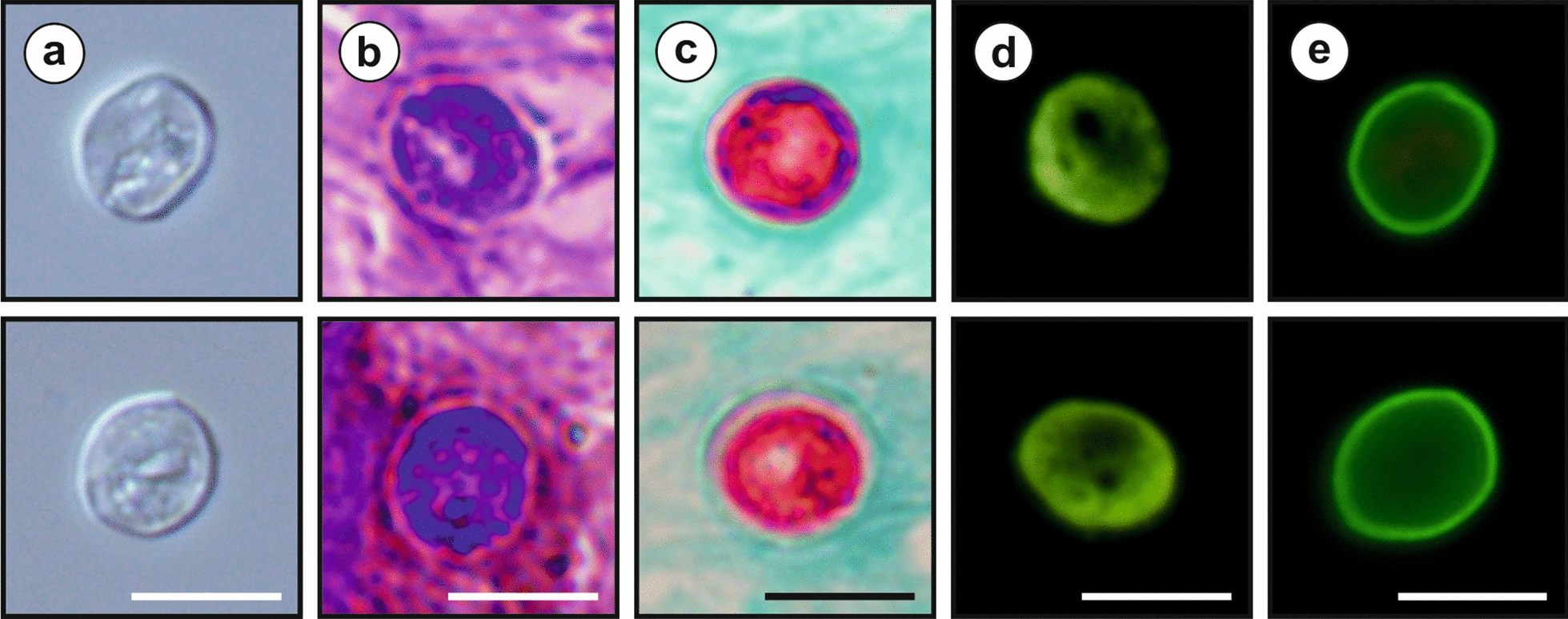
Oocysts of *Cryptosporidium* chipmunk genotype I **a** in differential interference contrast microscopy, **b** stained by aniline-carbol-methyl violet staining, **c** stained by Ziehl-Nielsen staining, **d** stained by auramine-phenol staining and **e** labeled with anti-*Cryptosporidium* FITC-conjugated antibody. Bars = 5 μm

The size of chipmunk genotype I oocysts obtained from a naturally infected human was not statistically different from those obtained from experimentally infected mice, ferrets and squirrels, measuring 5.64 (5.50–5.89) × 5.37 (4.86–5.60) μm with an index of 1.05 (1.01–1.14) (Table 1). Chipmunk genotype I oocysts in the fecal smears stained with ACMV, ZN and AP showed the typical *Cryptosporidium* staining characteristics (Fig. 8b–d), and intensity of staining did not differ from *C. parvum* oocysts used as controls (data not shown). Immunofluorescent reagents originally developed for *C. parvum* oocysts also reacted with antigens of chipmunk genotype I oocysts, resulting in positive immunofluorescent labeling (Fig. 8e).

**Table 1:** Size of *Cryptosporidium* chipmunk genotype I oocysts recovered from naturally (*) infected human (*Homo sapiens*) and experimentally (#) infected SCID (*Mus musculus*), Eurasian red squirrel (*Sciurus vulgaris*), eastern gray squirrel (*Sciurus carolinensis*) and domestic ferret (*Mustela putorius furo*)

Host (animal no.)	Length (μm) Range (mean ± SD)	Width (μm) Range (mean ± SD)	Length/width ratio Range (mean ± SD)
Human (CHIP_I)*	5.50–5.89 (5.64 ± 0.19)	4.86–5.60 (5.37 ± 0.17)	1.01–1.14 (1.05 ± 0.05)
SCID mouse (0)#	5.45–6.00 (5.62 ± 0.23)	4.82–5.64 (5.41 ± 0.24)	1.04–1.13 (1.06 ± 0.07)
SCID mouse (3)#	5.45–6.00 (5.62 ± 0.23)	4.82–5.64 (5.41 ± 0.24)	1.04–1.13 (1.06 ± 0.07)
Eurasian red squirrel (3)#	5.47–5.91 (5.57 ± 0.28)	4.89–5.63 (5.30 ± 0.19)	1.03–1.14 (1.04 ± 0.07)
Eastern gray squirrel (2)#	5.50–5.95 (5.61 ± 0.26)	4.90–5.60 (5.32 ± 0.22)	1.05–1.12 (1.04 ± 0.09)
Ferret (2)#	5.52–5.80 (5.60 ± 0.21)	4.81–5.69 (5.36 ± 0.23)	1.02–1.15 (1.05 ± 0.06)

Length and width of 100 oocysts from each isolate were measured under differential interference contrast at 1000× magnification, and these measurements were used to calculate the length-to-width ratio of each oocyst

### Infection site

**Fig. 9:**
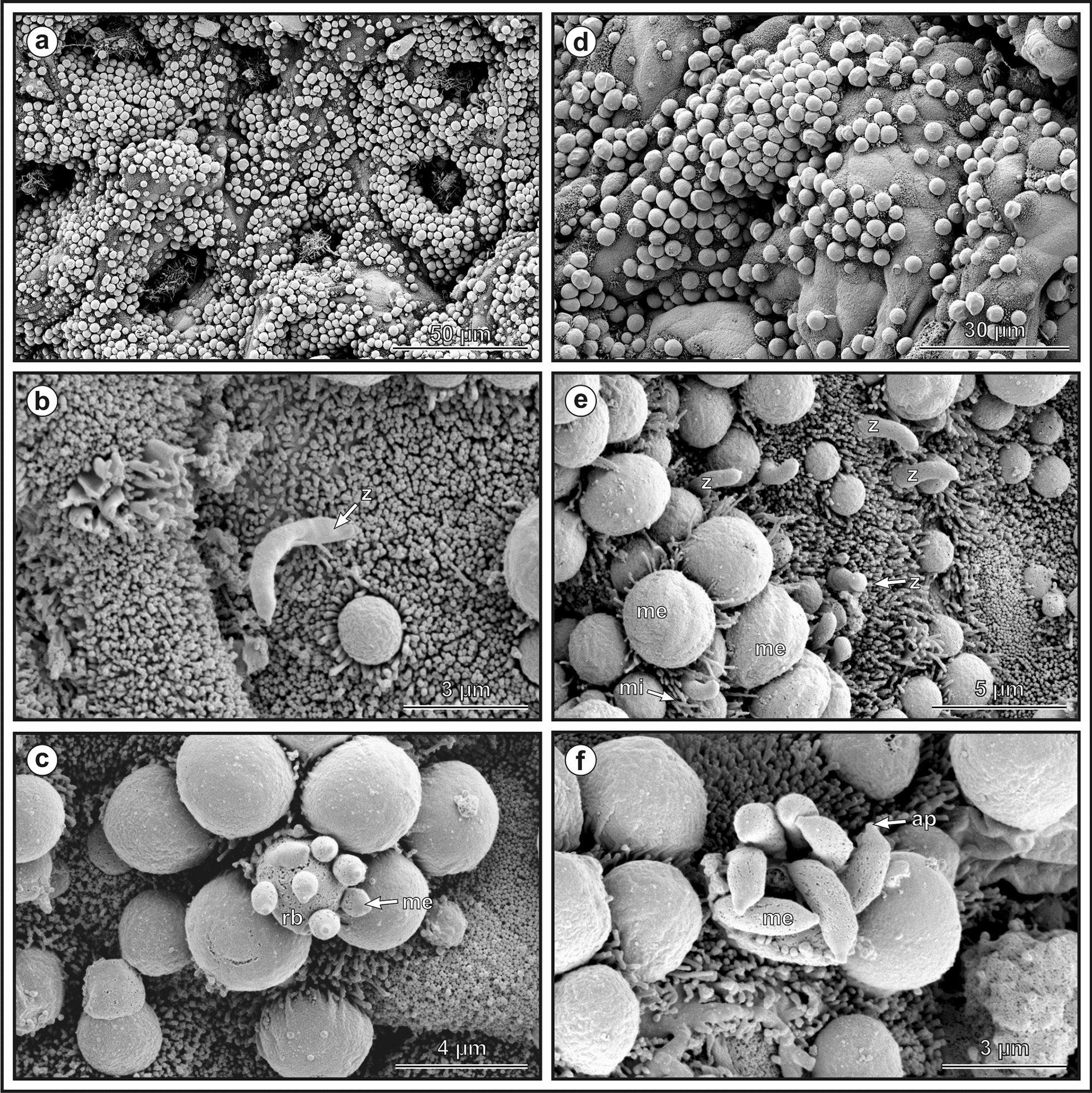
Scanning electron microphotograph showing developmental stages of *Cryptosporidium* chipmunk genotype I on cecal mucosal epithelium in experimentally infected Eurasian red squirrel (*Sciurus vulgaris*) killed 16 days post infection (DPI) (**a**–**c**) and SCID mouse (*Mus musculus*) killed 30 DPI (**d**–**f**). **a** and **d** Surface of cecum covered with developmental stages,** b** released zoite (z); **c** merozoites (me) budding from residual body (rb); **e** zoites invading host tissue (z) with formation of merozoites covered with parasitophorous sac (me) and surrounded by elongated microvilli (mi), **f** mature meront with fully developed merozoites (me) with recognizable apical part (ap). Scale bars included in each figure

**Fig. 10:**
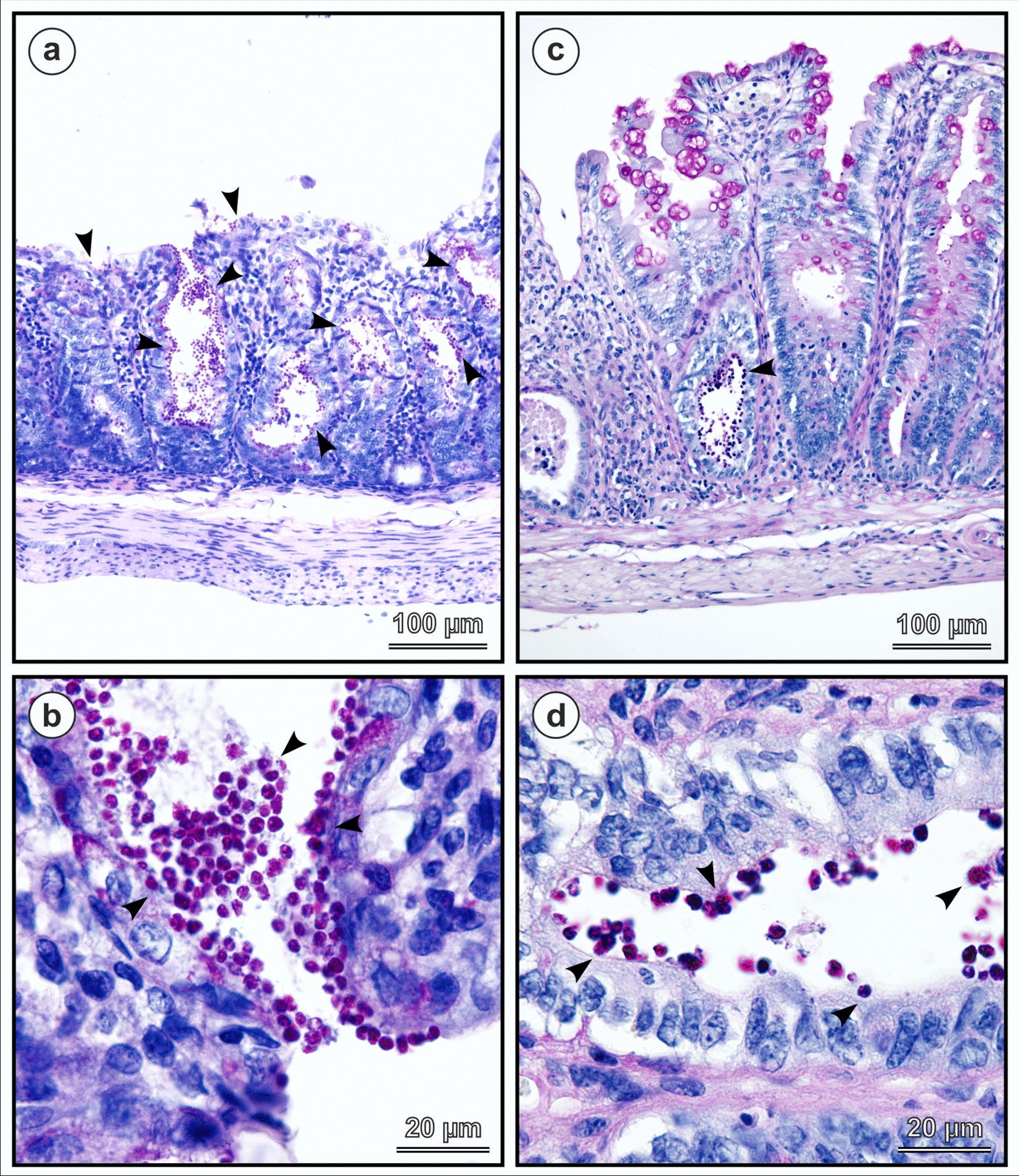
Histology sections of the cecum of Eurasian red squirrel (*Sciurus vulgaris*) (**a**, **b**) and SCID mouse (*Mus musculus*) (**c**, **d**) experimentally infected with *Cryptosporidium* chipmunk genotype I and killed 16 and 30 days post infection, respectively. Attached developmental stages indicated by arrowhead. Periodic acid-Schiff (PAS) staining. Scale bar included in each figure

Molecular, histological, SEM and TEM analyses showed the presence of chipmunk genotype I DNA and parasite developmental stages exclusively in the cecum of all susceptible hosts included in this study. Additionally, in SCID mice and Eurasian red squirrels, the proximal part of the colon was infected. The developmental stages covered almost the entire epithelial surface (Figs. 9 and 10).

### Morphology and morphometry of endogenous developmental stages

**Fig. 11:**
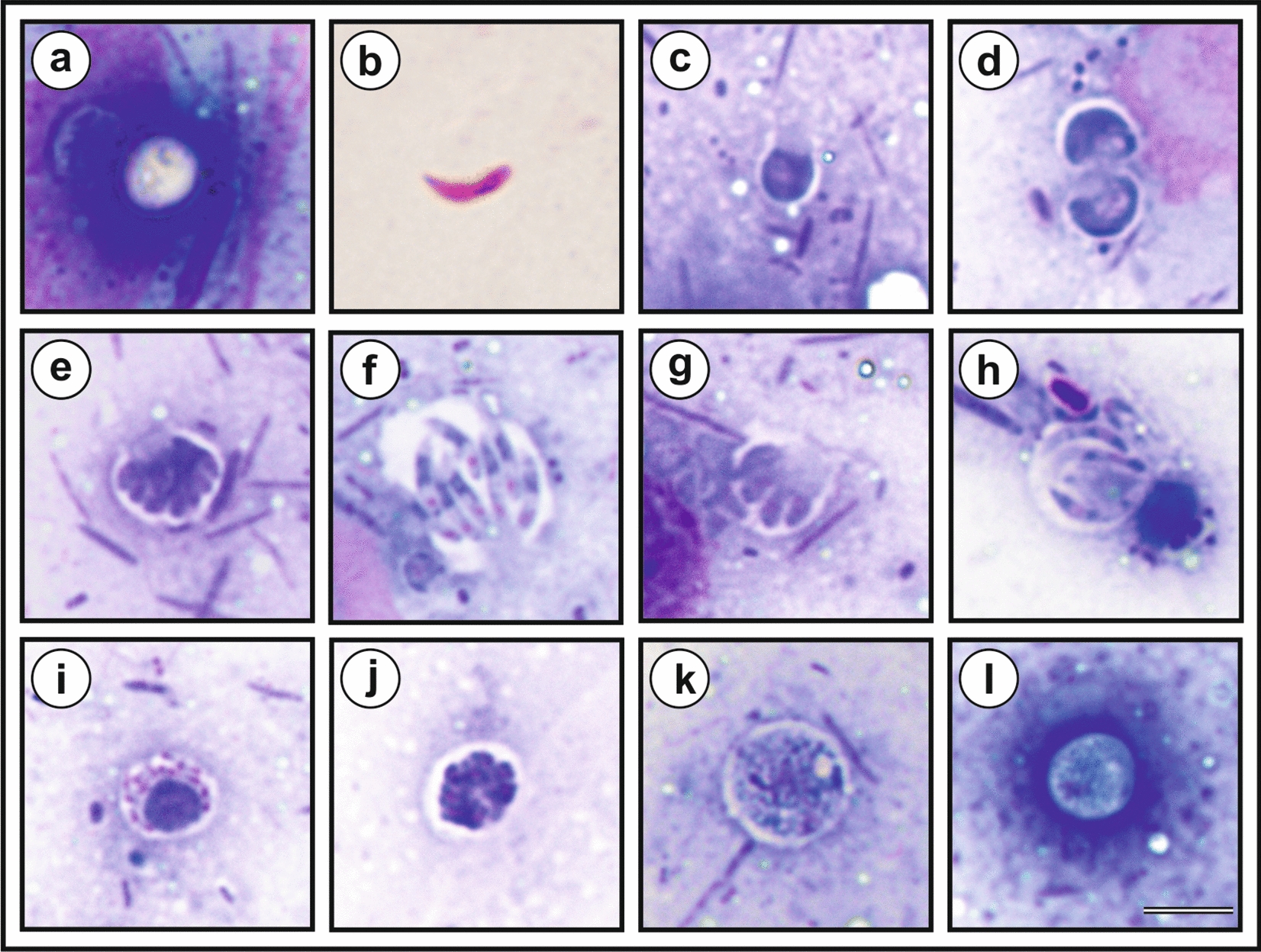
Developmental stages of *Cryptosporidium* chipmunk genotype I in mucosal smears obtained from the cecum of SCID mouse (*Mus musculus*) experimentally infected with 100,000 oocysts and killed 30 days post infection. **a** Oocyst; **b** sporozoite; **c**, **d** mononuclear trophozoite; **e** eight-nuclei meront; **f** merozoites from eight-nuclei meront; **g** four-nuclei meront; **h** merozoites from four-nuclei meront; **i** and **j** microgamont; **k** macrogamont and **l** zygote. Bar = 10 μm

It was almost impossible to identify developmental stages from SEM observations because most stages were covered with parasitophorous vacuoles. We were only able to identify stages with ruptured parasitophorous vacuoles. Early meronts with incompletely separated or fully developed merozoites and freely invading merozoites were observed. Using Wright-stained smears and analysis of TEM, we were able to determine all developmental stages. In the Wright-stained smears, the parasitophorous vacuoles surrounding the stages remained unstained, and the nuclei were stained pink (Fig. 11). Mononuclear trophozoites, transitional vegetative stages, were the most frequently observed stages, and their size was highly variable (Table 2).

**Table 2:** Size of developmental stages of *Cryptosporidium* chipmunk genotype I obtained from the cecum of SCID mouse (*Mus musculus*) experimentally infected with 100,000 oocysts and killed 30 days post-infection

Developmental stages	Length (μm) Range (mean ± SD)	Width (μm) Range (mean ± SD)
Oocyst	5.50–5.89 (5.64 ± 0.19)	4.86–5.60 (5.37 ± 0.17)
Sporozoite	4.67–5.92 (5.42 ± 0.41)	0.55–0.64 (0.61 ± 0.03)
Trophozoite	1.95–5.27 (3.26 ± 0.76)	1.56–4.71 (2.75 ± 0.67)
Eight-nuclei meront	4.49–6.42 (5.54 ± 0.52)	4.07–6.04 (4.91 ± 0.53)
Merozoite from eight-nuclei meront	3.76–6.29 (5.22 ± 0.62)	0.51–0.98 (0.73 ± 0.12)
Four-nuclei meront	4.18–5.28 (4.65 ± 0.47)	3.86–5.21 (4.40 ± 0.58)
Merozoite from four-nuclei meront	4.88–5.63 (5.42 ± 0.32)	0.51–0.92 (0.73 ± 0.17)
Macrogamont	4.78–8.16 (5.93 ± 0.68)	4.37–6.46 (5.42 ± 0.62)
Microgamont	4.53–5.42 (4.91 ± 0.32)	4.14–4.42 (4.28 ± 0.10)
Zygote	4.91–5.53 (5.29 ± 0.22)	4.16–5.11 (4.79 ± 0.30)

Measurements were obtained via SEM

**Fig. 12:**
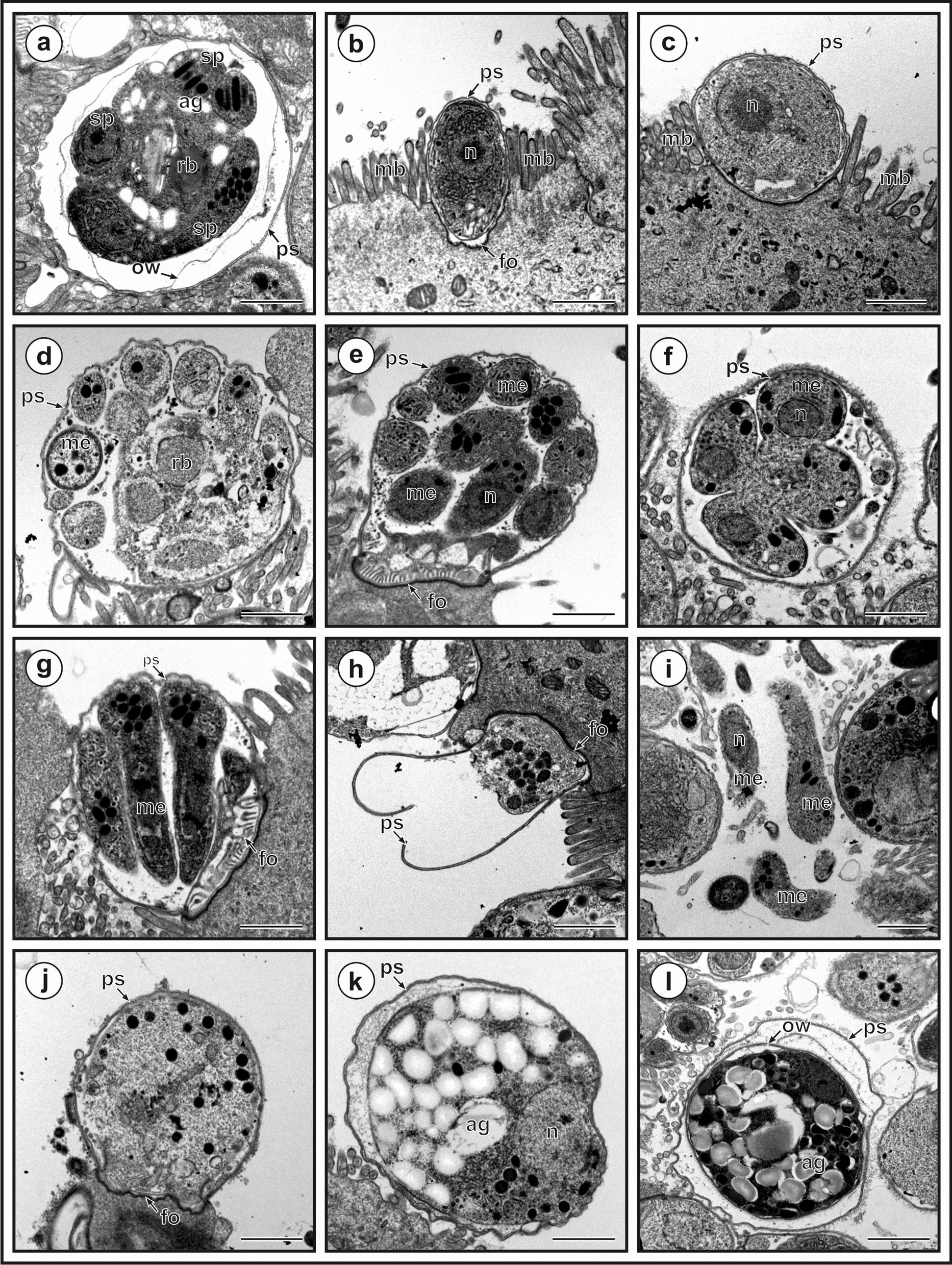
Developmental stages of *Cryptosporidium* chipmunk genotype I in transmission electron microscopy. **a** Oocyst with four sporozoites (s), residual body (rb) with amylopectin granules (ag), forming oocyst wall (ow) in parasitophorous sac (ps); **b** early trophozoite with one nucleus (n) inside parasitophorous sac (ps) and attached to microvilli border (mb) with feeding organelle (fo);** c** later trophozoite with one nucleus (n) inside parasitophorous sac (ps) and attached to microvilli border (mb); **d** early meront covered with parasitophorous sac (ps) with forming eight merozoites (me) connected to residual body (rb); **e** cross section of mature meront covered with parasitophorous sac (ps), fully developed eight merozoites (me) with visible nucleus (n) and connected to host cell by feeding organelle (fo); **f** cross section of early meront with forming four merozoites (me) with visible nucleus (n) and covered with parasitophorous sac (ps);** g** cross section of mature meront with fully developed four merozoites (me), covered with parasitophorous sac (ps) and attached to host cell by feeding organelle (fo); **h** empty parasitophorous sac (ps) attached to the host cell by feeding organelle (fo); **i** released merozoites (me) with visible nucleus (n); **j** early microgamont (mi) and attached to the host cell by feeding organelle (fo); **k** macrogamont covered with parasitophorous sac (ps) with foam-like appearance caused by amylopectin granules (ag) and visible nucleus (n); **l** zygotes with amylopectin granules (3), developing oocyst wall (1), parasitophorous sac (2). Bar = 1 um

Meronts with eight nuclei that became part of the forming merozoites were frequently observed, whereas meronts with four nuclei were rarely observed and differed from meronts with eight nuclei in that they had four nuclei from which four merozoites formed (Fig. 11e–h). Microgamonts were rarely observed and were easily recognized by 16 nuclei that became part of the forming microgametes (Fig. 11i–j). Macrogamonts were filled with oval amylopectin granules that gave the macrogamont a foamy appearance, making these stages easy to identify (Fig. 11k). A foamy structure was also observed in early zygotes, but it was less pronounced compared to the macrogamont stages (Fig. 11l). Zygotes and oocysts did not exhibit parasitophorous vacuoles. Oocysts were observed to be unstained and without significant structures. We were unable to distinguish between thin- and thick-walled oocysts. In the TEM analysis, the feeder organelle was clearly visible at most stages (Fig. 12). Early trophozoites were oval and became more spherical as they matured (Fig. 12b–c). Eight-nuclei meronts and four-nuclei meronts differed in the number of developing merozoites (Fig. 12d–g). TEM observation showed amylopectin granules not only in the macrogamont but also in the zygotes (Fig. 12k–l). Compared to the macrogamont, fewer amylopectin granules were present in the zygotes, and a new wall formation was visible (Fig. 12l). Compared to the Wright stain, four sporozoites were visible inside the oocysts around a residual body formed from amylopectin granules (Fig. 12a).

### Clinical signs and pathogenicity

In ferrets, clinical signs of cryptosporidiosis, vomiting and watery diarrhea occurred at 6–7 DPI. In infected SCID mice, a change in feces from solid globules to a paste-like consistency was observed from the 2nd week post infection, but no vomiting was observed. In one of the eastern gray squirrels, a slight apathy was observed at 7–8 DPI, manifested by reduced interest in the surroundings and food. The droppings in this individual had a pasty consistency during this period. Eurasian red squirrels quickly lost their condition and appetite for food from day 10 after infection. They spent most of the day in their shelter, were lethargic and did not respond to external stimuli-feeding, watering, handling or cage cleaning. Intestinal crypts were multifocally dilated with atrophy of epithelial and mucus cells. The formation of crypt microabscesses was locally observed. A multifocally to diffusely pronounced lymphoplasmocytic inflammatory infiltrate with admixture of smaller amounts of eosinophilic and neutrophilic granulocytes was present in the stroma of the intestinal mucosa. Reactive hyperplasia of submucosal lymphoid tissue/follicles was detected. Infected cells showed increased microcell elongation.

Based on the presented data, we propose *Cryptosporidium* chipmunk genotype I as a new species, with the species description presented below.

## Taxonomic summary

**Family Cryptosporidiidae Léger, 1911**

**Genus** ***Cryptosporidium*** **Tyzzer, 1907**

***Cryptosporidium mortiferum*** **n. sp.**

**Syn:** *Cryptosporidium* chipmunk genotype (W17), *Cryptosporidium* genotype W17, *Cryptosporidium* chipmunk genotype I.

**Type host:** Eastern chipmunk (*Tamias striatus*) [19]

**Other hosts:** Deer mouse (*Peromyscus* sp.) [19], American red squirrel (*Tamiasciurus hudsonicus*) [20], Eurasian red squirrel (*Sciurus vulgaris*) [21], striped field mouse (*Apodemus agrarius*) [43], Ussuri white-toothed shrew (*Crocidura lasiura*) [43], Pallas's squirrel (*Callosciurus erythraeus*) [24], human (*Homo sapiens*) [44].

**Type locality:** New York, USA.

**Type material:** Fecal smear slides with oocysts stained by ACMV and ZN staining (nos. MV1-5/34351 and ZN1-5/34351); scanning electron microscopy specimens of infected cecum (nos. SEM220/2017, SEM221/2017, SEM43/2020 and SEM44/2020) and colon (nos. SEM222/2017, SEM223/2017, SEM52/2019 and SEM53/2019); transmission electron microscopy specimens of infected cecum (nos. TEM220/2017, TEM221/2017, TEM43/2020 and TEM44/2020) and colon (nos. TEM222/2017, TEM2023/2017, TEM52/2019 and TEM53/2019); histological sections of infected cecum (nos. H220/2017, H221/2017, H43/2020 and H44/2020) and colon (nos. H222/2017, H223/2017, H52/2019 and H53/2019); gDNA isolated from fecal samples of naturally infected human (isolate CHIP_I) and experimentally (isolate 34351) infected Eurasian red squirrel (*Sciurus vulgaris*); gDNA isolated from cecum and colon of experimentally infected Eurasian red squirrel (isolates 35039 and 35041). All specimens are deposited at the Institute of Parasitology, Biology Centre of the Czech Academy of Sciences, Czech Republic.

**Site of infection:** Cecum, colon (present study).

**Prepatent period:** Range from 4 to 12 DPI (this study), depending on the host species.

**Patent period:** Range from a few days to many weeks or a fatal infection (this study), depending on the host species and its immune response.

**Representative DNA sequences:** Representative nucleotide sequences of *SSU* [OQ627025–OQ627029], actin [OQ632461–OQ632466], *HSP*70 [OQ632480–OQ632487], *TRAP-C1* [OQ632488–OQ632495], *COWP* [OQ632467–OQ632474] and *gp60* [OQ632475–OQ632479] genes are deposited in the GenBank database.

**ZooBank registration:** To comply with the regulations set out in Article 8.5 of the amended 2012 version of the International Code of Zoological Nomenclature (ICZN), details of the new species have been submitted to ZooBank. The Life Science Identifier (LSID) of the article is urn:lsid:zoobank.org:pub:1E90AC9B-DD71-4DF1-B554-F5AEDC82CD5A. The LSID for the new name *Cryptosporidium mortiferum* n. sp. is urn:lsid:zoobank.org:act:381F6044-C574-4F9D-8FC8-89BEABEB585B.

**Description:** The oocyst wall is smooth and colorless. The oocyst is composed of one spherical globule residuum, numerous small granules and four sporozoites. A suture is not noticeable. Sporulated oocysts measure 5.50–5.89 (5.64 ± 0.19) × 4.86–5.60 (5.37 ± 0.17) µm with a length-to-width ratio of 1.01–1.14 (1.05 ± 0.05) (Fig. 9).

**Etymology:** Since the infection caused by this species in Eurasian red squirrels is lethal, the species name *mortiferum* is derived from the Latin *mortifer*, meaning lethal.

**Differential diagnosis:** Oocysts of *C. mortiferum* are stained by ACMV and ZN staining methods and labeled with genus-specific antibodies targeting the *Cryptosporidium* oocyst outer wall antigenic sites, similar to other *Cryptosporidium* spp. (Fig. 9). Oocysts of *C. mortiferum* are larger than those of *C. parvum* (*T*2 = 320.42, *df1* = 2, *df2* = 69.00, *p* < 0.001) and *C. sciurinum* (*T*2 = 67.32, *df1* = 2, *df2* = 84.60, *p* < 0.001). The oocyst size cannot be used for species identification. *Cryptosporidium mortiferum* can be differentiated genetically from other *Cryptosporidium* spp. based on nucleotide sequences of *SSU*, actin, *HSP70*, *TRAP-C1*, *COWP* and *gp60* genes.

## Discussion

Infection with *C. mortiferum* in eastern gray squirrels under experimental conditions is usually asymptomatic and is associated with a minor infection followed by self-healing. This is consistent with the finding by Prediger et al. of a low infection intensity in naturally infected eastern gray squirrels [24]. In agreement with the findings of Bujila et al. [25], we observed massive infections with *C. mortiferum* in Eurasian red squirrels, which were accompanied by severe clinical signs of cryptosporidiosis and resulted in death of the individuals. Because Eurasian red squirrels infected with *C. mortiferum* restrict their movement, lose interest in foraging and remain in their burrows most of the time, infected Eurasian red squirrels would be difficult to capture in live traps used in field research. It is likely that only a small percentage of individuals infected with *C. mortiferum*, such as those in the early stages of infection, would be captured in field studies.

This work clearly demonstrated that *C. mortiferum* pathogenicity varies among host species and that the course of infection is impacted by the host species and the immune status. The infection was asymptomatic in immunocompetent laboratory mice. Consistent with previous studies examining the host immune response to *Cryptosporidium* infection, mice deficient in CD4+ or CD8+ lymphocytes showed a longer course of infection with *C. mortiferum* than immunocompetent individuals [45, 46]. However, the absence of CD4 or CD8 lymphocytes did not prove critical, and animals with this deficiency recovered from infection. As with infections with *C. parvum*, *C. proliferans* and *C. tyzzeri*, self-curing did not occur in SCID mice infected with *C. mortiferum* [6, 45, 47]. SCID and BALB/c mice may serve as a suitable laboratory model for the study of cryptosporidiosis affecting the cecum. The prepatent period of *C. mortiferum* was found to be different in different host species/strains. While ferrets and mice, with the exception of SCID mice, began shedding oocysts/specific *C. mortiferum* DNA at 4–5 DPI, the prepatent period was longer in squirrels, SCID mice and gerbils (7–14 DPI). A similar difference in the length of the prepatent period, depending on the host species, was observed in *C. proliferans* [6]. The intensity of *C. mortiferum* infection also varied depending on the species and immune status of the host. Similar differences were observed in other *Cryptosporidium* species, e.g. *C. alticolis* and *C. microti* infecting various species of voles, *C. apodemi* and *C. ditrichi* parasitizing *Apodemus* spp., *C. proliferans* infecting various rodents or *C. ornithophilus* infecting geese, cockatiels and chickens [6, 7, 48, 49].

Most species of intestinal *Cryptosporidium* for which tissue specificity has been described parasitize the small intestine, e.g. *C. parvum*, *C. myocastoris*, *C. scrofarum*, *C. hominis*, *C. tyzzeri*, *C. ryanae* and *C. ditrichi* [14, 48, 50–54]. Only a small proportion of species parasitize the colon, or in birds, the bursa of Fabricius, e.g. *C. suis* or *C. baileyi* [55, 56]. The development of *C. mortiferum* occurs exclusively in the cecum and anterior colon. It is the first *Cryptosporidium* species in mammals to prefer this part of the intestine. A similar localization has already been described in *Cryptosporidium avium* and *C. ornithophilus* parasitizing birds [7, 17].

This study also described exogenous and endogenous developmental stages in *C. mortiferum*. The life cycle of *C. mortiferum* does not differ from that of previously described *Cryptosporidium* species. Consistent with studies that have examined the life cycle in vivo, we found no evidence for the occurrence of extracellular developmental stages described in cell-free cultures [57–59]. The oocysts of *C. mortiferum* measured 5.64 × 5.37 μm, which is similar in size to a previously published isolate of chipmunk genotype I (5.8 × 5.4 μm) found in Eurasian red squirrels [21]. The oocysts of this species are slightly larger than those of *C. sciurinum* (5.54 × 5.22 μm), *C. ubiquitum* (5.04 × 4.66 μm) and *C. parvum* (5.2 × 4.9 μm), which also have been detected in tree squirrels [8, 24, 60]. However, the differences in oocyst size are so small that they cannot be used for differential diagnosis among *Cryptosporidium* species by routine microscopy. Similar to previous studies, we were unable to distinguish between thin- and thick-walled oocysts, if present in this species [6, 61]. In agreement with the study by Holubová et al. [62], most of the observed developmental stages visualized by Wright staining were surrounded by a parasitophorous vesicle that appeared as an unstained aureole. In agreement with the same authors, mononuclear trophozoites and eight-nuclear merozoites were most frequently detected, while four-nuclei merozoites were rarely found with the Wright stain and TEM. In a recent in vitro study of *C. parvum*, English et al. [63] showed that the abundance of four-nuclear merozoites was low and that they were not required for microgamont and macrogamont formation. In addition, microgamonts were rarely found compared to macrogamonts [62, 63]. Examination of SEM showed elongation of microvilli of cells parasitized by developmental stages of *C. mortiferum*. Similar elongation was previously observed in coypu infected with *C. myocastoris* [53], SCID mice infected with *C. parvum* [64] and rats infected with *C. occultus* [15]. Borowski et al. [59] also reported the elongation of microvilli of cells infected with *C. parvum* in an in vitro model system.

Phylogenetic analyses at *SSU*, actin, *HSP70*, *TRAP-C1*, *COWP* and *gp60* loci confirmed previously published data showing that *C. mortiferum* is genetically distinct from other species within the genus *Cryptosporidium* and represent a separate species. At all loci, *C. mortiferum* formed a separate clade within the group of intestinal *Cryptosporidium* spp. and close to *C. viatorum*. At *SSU*, actin, *HSP70*, *TRAP-C1* and *COWP* loci, the pairwise distances between *C. mortiferum* and *C. viatorum* (0.005, 0.011, 0.007, ND and 0.013, respectively) were similar to those between *C. mortiferum* and *C. sciurinum*, which is a major *Cryptosporidium* species in Eurasian red squirrels (0.004, 0.012, 0.008, 0.016 and 0.013, respectively) and greater than those between *C. hominis* and *C. parvum* (0.002, 0.005, 0.003, 0.006 and 0.007, respectively) and between *C. hominis* and *C. cuniculus* (0.003, 0.001, 0.001, ND and ND, respectively).

To date, *C. mortiferum* has only been detected in the USA [44], Italy [21], Sweden [65] and South Korea [43], with most reported detections coming from the USA. The occurrence of *C. mortiferum* in Italy is related to the presence of introduced eastern gray squirrels, which are one of the natural hosts of this parasite. Sweden harbors only Eurasian red squirrels, and the authors suggest that these animals are natural hosts for *C. mortiferum* in Sweden [23]. Therefore, it is possible that *C. mortiferum* spread through the Eurasian red squirrel population and was introduced into northern Europe. However, studies in Central Europe have not shown that *C. mortiferum* is present in native squirrel populations in the Czech Republic and Slovakia, and we have no evidence of spread [8]. Therefore, the possibility that *C. mortiferum* was introduced to Sweden by other means cannot be excluded. This question needs further investigation. Similarly, we have no explanation for the isolated occurrence of *C. mortiferum* in the Ussuri white-toothed shrew in Korea. Guo et al. [34] showed two geographic clusters within the *C. mortiferum gp60* group (the XIVa subtype family). Samples from New York, Maine and Vermont, the three Northeastern US states, formed a common cluster, whereas samples from Minnesota and Wisconsin, the two Midwestern states, and Sweden formed another cluster [34]. All isolates obtained from naturally infected squirrels in Italy, as well as the CHIP_I isolate used in this study, belong to the Northeastern US group. These results show that, as in the Guo et al. [34] study, two separate geographic clusters within *C. mortiferum gp60* group also occur in Europe. Similarly, previous studies showed differences in geographic and host distribution of *gp60* families of *Cryptosporidium* spp. For example, *C. tyzzeri* family XIa exclusively infects *Mus musculus musculus* at the eastern part of the European mouse hybrid zone, while family XIb occurs only in the western part, infecting *M. m. domesticus* [47]. Similarly, *C. hominis* family Ib appears to predominate in most studies in Europe, North America and high-income countries in Oceania compared to other regions [66]. Due to the high pathogenicity of *C. mortiferum* to Eurasian red squirrels, which most likely complicates its detection in field research, and the lack of studies focusing on the occurrence of *Cryptosporidium* in squirrels in Europe, it is not possible to assess the occurrence and distribution of different *gp60* groups within the European red squirrel population.

## Conclusions

This study confirms that *Cryptosporidium* chipmunk genotype I is biologically and genetically distinct from all currently recognized species of the genus *Cryptosporidium*. The results support the description of this *Cryptosporidium* as a separate species, which we propose to name *Cryptosporidium mortiferum* n. sp. Transmission studies demonstrate the high pathogenicity of this species to Eurasian red squirrels. The rapid progression of the infection, resulting in death of the infected individual, is one explanation why *C. mortiferum* is detected in Eurasian red squirrels at low prevalence, although it is infectious to them. In agreement with previous studies, Eurasian red squirrels are considered an important source of *C. mortiferum* infection for humans.

## Data Availability

All type material and datasets on which the conclusions of the manuscript rely are stored at the Institute of Parasitology, Biology Centre, Czech Academy of Sciences, České Budějovice, Czech Republic. Representative nucleotide sequences generated in this study were submitted to the GenBank database under the accession numbers OQ627025–OQ627029, OQ632461–OQ632495.

## Abbreviations

ACMV: Aniline-carbol-methyl violet
AP: Auramine phenol
BSA: Bovine serum albumin
BC CAS: Biology Center of the Czech Academy of Science, Czech Republic
COWP: *Cryptosporidium* Oocyst wall protein
DIC: Differential interference contrast
DNA: Deoxyribonucleic acid
DPI: Days post-infection
FITC: Fluorescein isothiocyanate
gDNA: Genomic DNA
gp60: 60-KDa glycoprotein gene
HE: Hematoxylin and eosin
HSP70: 70-KDa heat-shock protein
ICZN: International Commission on Zoological Nomenclature
IFA: Immunofluorescence assay
LSID: Life Science Identifier
ND: Not defined
NJ: Neighbor-joining
ML: Maximum likelihood
OPG: Oocysts per gram
PAS: Periodic acid-Schiff
PBS: Phosphate buffered solution
PCR: Polymerase chain reaction
Taq polymerase: *Thermophilus aquaticus* Polymerase
TRAP-C1: Thrombospondin related adhesive protein of *Cryptosporidium*-1
SD: Standard deviation
SEM: Scanning electron microscopy
SCID: Severe combined immunodeficiency
SSU: Small subunit of rRNA
TEM: Transmission electron microscopy
ZN: Ziehl–Neelsen
